# Opportunities and risks of large language models in psychiatry

**DOI:** 10.1038/s44277-024-00010-z

**Published:** 2024-05-24

**Authors:** Nick Obradovich, Sahib S. Khalsa, Waqas U. Khan, Jina Suh, Roy H. Perlis, Olusola Ajilore, Martin P. Paulus

**Affiliations:** 1 Laureate Institute for Brain Research, Tulsa, OK USA; 2 Oxley College of Health and Natural Sciences, University of Tulsa, Tulsa, OK USA; 3 Institute of Health Policy, Management and Evaluation, University of Toronto, Toronto, ON Canada; 4 Microsoft Research, Redmond, WA USA; 5 Center for Quantitative Health, Massachusetts General Hospital, Boston, MA USA; 6 Department of Psychiatry, Harvard Medical School, Boston, MA USA; 7 Department of Psychiatry & Behavioral Health, University of Illinois Chicago, Chicago, IL USA

**Keywords:** Business and industry, Technology

## Abstract

The integration of large language models (LLMs) into mental healthcare and research heralds a potentially transformative shift, one offering enhanced access to care, efficient data collection, and innovative therapeutic tools. This paper reviews the development, function, and burgeoning use of LLMs in psychiatry, highlighting their potential to enhance mental healthcare through improved diagnostic accuracy, personalized care, and streamlined administrative processes. It is also acknowledged that LLMs introduce challenges related to computational demands, potential for misinterpretation, and ethical concerns, necessitating the development of pragmatic frameworks to ensure their safe deployment. We explore both the promise of LLMs in enriching psychiatric care and research through examples such as predictive analytics and therapy chatbots and risks including labor substitution, privacy concerns, and the necessity for responsible AI practices. We conclude by advocating for processes to develop responsible guardrails, including red-teaming, multi-stakeholder-oriented safety, and ethical guidelines/frameworks, to mitigate risks and harness the full potential of LLMs for advancing mental health.

## Lay Summary

Imagine a conversation with an advanced computer program that could help doctors understand and treat mental health better. In this paper, we discuss using such advanced programs, called Large Language Models, in psychiatry. While they might offer quicker, tailored help and improve how mental health services work, there are many big questions left to answer about how to use this technology safely and ethically, to benefit patients without causing unintended harm.

## Opportunities and risks of large language models in psychiatry

Take a Deep Dive into this article

## Introduction

New technologies can profoundly affect mental health research and psychiatric treatment [1]. For example, mobile computing devices and telehealth software offered unprecedented opportunities to improve access to care (e.g., interacting with a psychiatrist or psychotherapist via a smartphone), monitor clinical status (e.g., using a tablet to track personal outcomes), and collect clinically relevant data (e.g., allowing a smartwatch to record blood pressure to gauge stress levels). These tools offer advantages including convenience, anonymity, affordability, and the ability to reach underserved populations. These advantages also reduce the costs [2] and logistical challenges of traditional research methods. Currently, marked advances in the sophistication of large language models (LLMs) [3] may be poised to alter population mental health, mental health research, and mental healthcare practices. To understand the implications of this technological advance, we provide an overview of LLMs and describe some prominent applications for mental health. Additionally, we discuss the opportunities and risks LLMs might pose to the public’s mental well-being and to healthcare professionals’ efforts.

### Function and use of LLMs

The term artificial intelligence (AI) denotes both (i) a scientific discipline studying how to build and understand computer systems that can complete endeavors deemed difficult because of the intelligence they seem to involve and (ii) those computer systems themselves [4]. As a scientific discipline, AI contains subfields that focus on the development of mechanical devices that can accomplish tasks for humans via movement, computing systems that can extract information from images, and software that can construe information in natural language for the purpose of devising further information for a user. As computer systems exhibit intelligence, AI possesses diverse abilities to solve problems, devise complex plans, integrate diverse technical information, and generate content (e.g., text, images, and audio). AI tools that generate content such as text and images have received attention over the past year due to the release of systems that make these tools easily accessible online (e.g., by February 2023, OpenAI’s suite of AI tools reached 100 million active users in 2 months) [5].

While the remarkable performance of the most advanced AI tools is quite novel, they rely on technical infrastructure and training processes—namely, artificial neural networks and machine learning—that have existed for decades [6–9] but rapidly progressed recently with the expansion of computing power that allows them to operate at substantially larger scale [10]. Artificial neural networks [8] are systems of weighted mathematical functions that slightly resemble neurons in the brain, ‘firing’—that is, producing an output—when the value of their inputs reaches some threshold value [8, 11, 12]. Machine learning constitutes a wide range of statistical methods [13], including those for estimating parameters and specifying connection structures in artificial neural networks [12]. Progress in these methods has occurred in recent decades [6–14], particularly with the development of simplified network architecture (i.e., transformers) capable of rapidly and efficiently conducting parallel processing of sequential data such as text [15].

From these methods, models with impressive language capacities have emerged (e.g. ChatGPT-4 from OpenAI [16]). Humans can hold advanced conversations with LLM partners via a standard interchange: a user supplies a ‘prompt’ to the system in the form of a written statement and the LLM generates an output in the form of the most probable completion of the prompt. Prompts typically consist of instructions, context, input data, and output indicators, although these categories can be arbitrary (e.g. as in few-shot learning). Once a user supplies a prompt as input, the system breaks the prompt’s words into fragments that are passed through the model’s neural network to produce an output that it returns to the user [12]. This output is the model’s estimate of the most probable continuation of the input that the user supplied.

Because of their myriad cross-domain applications and public accessibility, LLMs have rapidly entered widespread use. Their abilities now figure in tools ranging from online search assistants to social bots, from essay-writing tools to clinical therapeutic assistants.

### Potential implications of LLMs for mental health

The application of LLMs as general-use technological tools has already yielded anticipated and unanticipated consequences [17]. LLMs notably excel in efficiently acquiring information, condensing content, and tackling reasoning-intensive problems [3]. In the context of mental healthcare, LLMs could conceivably quickly assimilate patient data, summarize therapy sessions, and aid in complex diagnostic problem-solving, thus potentially saving users and health systems both significant time and effort. Furthermore, when integrated into roles as personal assistants, LLMs can help individuals keep better track of their priorities and tasks, thus helping them achieve objectives in their daily lives. In this section, we discuss implications that include equitable access to the tools, manners in which LLMs may reshape mental healthcare systems, certain population mental health risks posed by the tools, and a framework for considering and evaluating such risks.

#### Equitable access to tailored LLMs

While ‘out-of-the-box’ LLMs might perform adroitly at mental healthcare-related tasks, they can also be further aligned to such tasks via approaches such as fine-tuning (i.e., trained specifically using additional data after its initial training), in-context-learning, or retrieval augmented generation, among other techniques to enhance task-specific performance (i.e., allowing longer prompts). Such models when fine-tuned for mental healthcare could, for example, offer a detailed list of therapeutic resources when asked how to manage a particular phobia. However, while the capacity to process information is increasing, processing such information at scale can be quite costly in terms of computing power and development resources, and therefore not all providers or institutions may benefit equally from the ability to fine-tune LLMs. This could become particularly problematic in mental health contexts, where accuracy in understanding the nuanced expressions of symptoms, emotions, and self-reported experiences is paramount.

#### Managerial and process-related considerations

LLMs have the potential to reshape health system managerial activity and work processes [18]. In a recent example of this possibility, Jiang et al. [19] demonstrated how an LLM trained on clinical notes could successfully predict patients’ readmission, in-hospital mortality, comorbidity index, length of stay, and insurance denial in the NYU Langone Health System. On its face, the findings relate solely to prognosis, but viewed more broadly the results provide grounds for “systems solutions”—redesigns of the hospital’s management and processes [20] that enhance decision-making via acting on the implications of AI predictions [18]. Operational decisions about personnel schedules and equipment needs could draw on the readmission and length of stay predictions reported in the paper—that is, a prediction that a wave of patients might require readmission or longer stays could activate increases in staffing levels and hospital material orders. Likewise, financial and accounting decisions could follow from bulk predictions of insurance denial: predictions of denial could galvanize steps to redirect funds from other parts of the organization or gather charitable resources to cover care a patient cannot afford. From simple predictions made by those five outcomes and additional training relating to the administrative implications of those outcomes, the LLM could automate managerial decisions within the hospital system, as research has discussed in other contexts [18]. Such possibilities portend a future in which not only clerical tasks become automated but also certain administrative ones.

#### Population mental health risks of LLMs

One significant risk associated with the general capacities of LLMs is that they not only make human labor more efficient, they can substitute for that labor [21]. If such labor substitution occurs rapidly and *en masse*, population mental health could experience declines mirroring those from prior economic downturns; clinical communities should prepare for such downturns. Another potential impact of LLMs on population mental health relates to the ease of developing conversational applications via the models. Due, perhaps, to the development of these tools for chat applications that require a warm tone, such as customer service, production LLMs typically exhibit a distinctive attribute: they provide pleasant interactions. On its face, this pleasant tone appears beneficial. Having an approachable AI with which to converse might seem to add a friend, albeit simulated, to one’s life and it might make day-to-day tasks more pleasing. But a fluid conversation partner that recalls detailed aspects of your life, coupled with a warm tone, runs the risk of sparking a feedback loop whereby users substitute human-to-human socialization with human-to-machine interaction. Innovative research studying the iterative rollout of Facebook across U.S. college campuses now provides reason to believe that social media may have produced unintended and costly consequences for individual well-being [22], and LLMs raise similar—if not greater—potential for such outcomes [23]. Moreover, a subset of adults can experience the onset or worsening of depression with greater social media use [24]. This risk grows because, much like social media, some AI chat applications may be optimized for user engagement, thus encouraging prolonged interaction. However, unlike social media, LLMs interact in ways that involve instantaneous conversation, the provision of information users sought for its value in professional or leisure purposes, and extensive opportunities for customization that can further enhance engagement.

It is important to recognize that the impact of LLMs on individual well-being can differ from person to person or may depend on how they are used, much like the effects of social media. Previous researchers not only have produced AI models with the explicit goal of enhancing users’ peer-to-peer empathy, but they have also demonstrated that the use of such models via human-AI collaboration actually can increase user empathy over time [25]. Consequentially, a substantial outcome involves the possibility of augmenting real-world or online human interactions with LLM interactions due to feelings of isolation or loneliness, which are currently at an all-time high [26]. For example, LLMs can be helpful for people looking to increase their social activities locally (e.g. via aiding internet searching for such options). On the converse, those suffering from social anxiety, social disconnection, or loneliness might be more inclined to use LLMs as a substitute for human-to-human socialization. These risks and benefits are ultimately empirical questions that require further study and consideration of (1) the situations in which LLMs are used, (2) the alternatives available to any given individual, (3) the principles guiding LLMs’ responses to queries, and (4) the degree of monitoring of the consequences of interactions with LLMs.

#### A framework for risk assessment

Scholars [27] have proposed a framework to better examine the broad social impact of LLMs, which includes grievous problems such as embedding biases, reinforcing stereotypes, violating privacy, and exacerbating inequalities, among other issues. LLMs – similar to other artificial intelligence and machine learning tools [28] – are subject to bias [29], both stemming from the content on which they are initially trained and the subsequent reinforcement input designed to shape their behavior. As such, practitioners should be cognizant of potential biases in the models that may manifest in unexpected ways in practice. Added to this are concerns about the potential for nonsensical or fanciful responses by LLMs to produce misinformation or confusion among users, particularly those users with pre-existing thought disorders. Given the complex determinants of population mental health, such broad-scale social implications [17] require holistic evaluation.

**Fig. 1:**
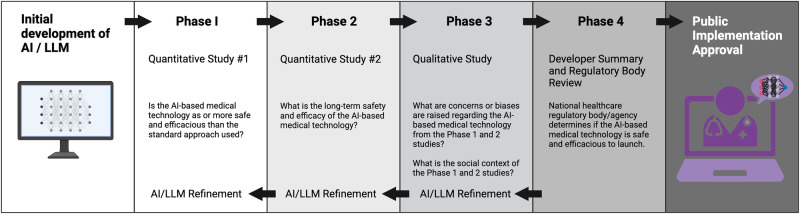
Introducing the Biological, Psychological, Economic, and Social (BPES) Framework: an innovative model for evaluating AI-based medical systems. Adapted from the pharmaceutical industry’s multi-phase clinical drug trial model, the BPES Framework is a translational scientific approach that can be applied to any regulatory or non-regulatory guideline that assesses an AI-based medical system’s safety and efficacy, before it is launched to the public. Figure created with Biorender.com.

In the medical realm, there are many existing and proposed frameworks/guidelines for developing AI systems that include LLMs. However, none to our knowledge provide a sufficiently clear understanding of what safety parameters should be assessed and whose safety should be considered when determining whether an AI medical application, tool, or device is safe to launch [30–33]. Moreover, current frameworks/guidelines often lack a surveillance component to monitor and identify the long-term risks and benefits of AI medical systems. One approach that mitigates these concerns is the Biological-Psychological, Economic, and Social (BPES) framework [34]. This innovative framework applies aspects of the biopsychosocial model used in psychiatry while providing a descriptive approach that guides developers and regulatory agencies on how to assess whether an AI medical system is safe to launch (Fig. 1). More specifically, it asks developers to demonstrate the safety of their AI technology for multiple stakeholders (patients, healthcare professionals, industry, and government healthcare agencies) while using the BPES domains as parameters [34]. Another contribution of the BPES framework is its compatibility with and ability to provide context for all existing and future AI medical regulatory and non-regulatory frameworks/guidelines, including red-teaming approaches, that are concerned with human safety and AI product/system efficacy [34]. Although potentially useful, one aspect this model does not comprehensively address is the need for human-centered, iterative, participatory design prior to randomized trial conductions; such considerations could be incorporated in future iterations.

The BPES framework, or others like it, could also be nested within broader efforts to evaluate “Software as a Medical Device” (SaMD). For example, the International Medical Device Regulators Forum (IMDRF [35]) is a global group of medical device regulators have begun expanding their harmonizing efforts to include SaMD and has established a working group on artificial intelligence/machine learning [36] focused on promoting the development of safe and effective practices. However, in current practice, effective regulation remains an aspiration rather than a reality.

### Opportunities of LLMs for psychiatric care and research

LLMs [37] hold marked potential for mental healthcare and research [38], primarily due to the central role language plays in the description, manifestation of, and treatment for mental health disorders. [39] A straightforward application of LLMs in mental healthcare might focus on the status assessment of an individual’s mental health or the use of verbal/language-based interventions to change this state. LLMs could thus potentially help to assess mental illness severity, suggest possible diagnoses, generate treatment plans, monitor the effect of an intervention, provide risk assessment indicators (e.g., for recurrence or relapse of a condition), and offer evidence-based suggestions for when an intervention is no longer needed. For instance, Galatzer-Levy et al. [40] examined whether an LLM without finetuning, yet “explicitly trained on large corpuses of medical knowledge,” could project an individual’s psychiatric functioning accurately based on patient and clinical documentation. The team found that, indeed, the LLM succeeded in this task: they could not reject the null hypothesis that the LLM performed the same as human clinical assessors [40]. Such findings underscore the fact that LLMs have demonstrated potential in interpreting verbal information to infer underlying affect, cognition, and diagnosis, providing a novel approach to assessing an individual’s mental health [41].

In another investigation of the clinical utility of therapy chatbots, a recent study described the development of LUMEN, a problem-solving treatment therapy coach that utilized the natural language understanding and natural language processing features of the Amazon Alexa platform. Participants were randomized to 8 sessions with LUMEN or a waitlist control arm with functional neuroimaging, and clinical and behavioral assessments conducted before and after treatment. This pilot study found that compared to the waitlist control group, participants who received LUMEN showed increases in task-evoked dorsolateral prefrontal cortex (DLFPC) activity that correlated with improvements in problem-solving skills and reductions in the avoidant coping styles. Furthermore, there were modest effects for reductions in depression and anxiety symptoms in the LUMEN group compared to the control group [42]. Although this study demonstrated evidence that the use of therapy chatbots might reduce symptoms of internalizing disorders, it was conducted in a small sample of participants with mild-to-moderate depression and/or anxiety. Further work is needed to determine whether such digital mental health interventions might be appropriate or effective for patients with greater clinical symptom severity, or at population-level scales.

These results highlight just how quickly the technology surrounding language models is evolving. For example, with minimal instruction and data from a problem-solving treatment manual, a version of LUMEN was created with ChatGPT-4 in under an hour (by O.A. and colleagues on November 28, 2023). Beyond the advantage of speed, another benefit of using an LLM is that users can interact with the chatbot more naturally and can personalize the style and tone of the conversational interface, to accomplish tasks that include identifying potential mental health conditions, determining the causes of those conditions, recognizing emotions in conversations, and understanding the relationship between environmental events and emotional responses [43]. However, a major disadvantage – aside from the limitation that the chatbot is derived from a specific region and culture – is the lack of control and constraints on how the LLM-derived chatbot can and will respond. Notably, the performance of ChatGPT has been found to be sensitive to different prompting strategies and emotional cues [44]. Thus, current models have limitations [45], including unstable predictions and sensitivity to minor alterations in prompts [44]. Future development in this domain should consider approaches to allow for more freeform user input while restricting chatbot output to ensure treatment fidelity.

These applications of LLMs in clinical settings are a direct consequence of the models’ powerful capacity for natural language processing (NLP). For example, LLM-based NLP models can detect medication information from clinical notes, generate and process psychiatric clinical narratives, and extract clinical concepts and associated semantic relationships from text [46, 47]. Such abilities to produce thematically accurate texts from automated transcriptions of clinical interactions may be particularly useful to the psychotherapeutic setting, potentially enabling clinicians to focus more attention on patients and less on real-time or post-hoc clinical note-taking. Further, LLM-driven evaluations of textual information are increasingly being applied to healthcare scenarios like perinatal self-harm, suicide attempts, HIV risk assessment, and delirium identification [43]. For example, LLMs have generated suicide risk predictions using health records data [48]. Others have demonstrated the cost-effectiveness and improved recovery rates associated with using a conversational AI solution for “referral, triage, and clinical assessment of mild-to-moderate adult mental illness” [49].

LLMs also have the potential to enhance psychotherapy by powering chatbots that provide evidence-based interventions. Such chatbots have demonstrated significant potential in the realm of mental health applications, providing a range of psychological interventions such as cognitive behavioral therapy [50], acceptance and commitment therapy [51], and positive psychology skills [52]. Clinicians have used them to assist patients with various conditions, including panic disorder [50], hypertension [53], and cancer [52]. They have even proven beneficial in postoperative recovery scenarios [51], and have also enhanced knowledge and skills related to health management [54]. Chatbots can deliver interventions at the user’s convenience, track progress, send reminders, and provide real-time feedback, perhaps enhancing adherence to treatment and self-management practices in the process. Moreover, they can potentially reduce healthcare costs by addressing minor health concerns that do not necessitate a doctor’s visit. However, research has raised concerns that these chatbots need better domain-specific training data, fine-tuning by expert clinicians, and targeting toward more “skilled” end-users [55]. Such chatbots also should be subject to efficacy trials to determine whether or not they actually improve clinical outcomes. Further, the degree to which both users and clinicians support the use of chatbots in clinical settings is worth additional scrutiny.

Another emerging application of LLM is clinical decision support, sometimes described more hyperbolically as “precision medicine”. Even LLMs trained on broad corpora (i.e., not specific to medicine) encode large amounts of medical knowledge, recognizing medications, indications, and adverse effects, and they can even demonstrate physician-level performance on board examinations, with the highest scores for psychiatry [56]. For example, one initial investigation applied ChatGPT-4 without fine-tuning or augmentation to suggest potential next-step antidepressant treatments [57] using a set of previously validated clinical vignettes. That study found that an optimal medication was identified ~3/4 of the time. On the other hand, in nearly half of the vignettes, the model also identified a potentially contraindicated or suboptimal treatment option.

More recently, a follow-up study examined the application of an LLM augmented with excerpts from clinical treatment guidelines for bipolar depression. Rather than applying a standard retrieval-augmented generation approach, this model simply incorporated key guideline principles in the prompt itself. This approach resulted in the selection of an expert-identified next-step treatment option twice as often as the unaugmented model and outperformed a small sample of community clinicians [58]. Notably, while the rate of selection of potentially contraindicated or less preferred options was diminished in the augmented model, such options appeared in approximately 1 in 10 vignettes, highlighting the need to apply such models with care. Prior work using clinical dashboards also underscored the capacity for psychiatric decision-support tools to lead clinicians astray [59].

LLMs may also prove to be valuable in formulating psychiatric research questions. Currently, researchers must analyze a growing volume of information to formulate hypotheses and discern the constellation of factors contributing to an individual’s mental health disorder for effective treatment research planning. In comparison, LLMs can rapidly and inexpensively generate—or provide feedback on—themes, questions, and proposed mechanisms at the level of disorders, while identifying possible factors underlying a particular individual’s mental health condition and formulating an individualized treatment plan [60]. Although this rapidly developing field is currently in its infancy, lessons learned from other areas of clinical science may be instructive. Thus, a phased approach for how to develop AI systems for use in mental health (e.g., one akin to the Phase I – IV process for evaluating new pharmacological agents) is likely to be of considerable value.

### Risks of LLMs for psychiatric care and research

The application of LLMs in mental health research and care, while promising, is not without potential challenges [61]. First, the unpredictability and non-deterministic nature of some LLMs’ output causes concern in clinical settings. Like humans, these models can occasionally produce factually incorrect answers, make errors in simple arithmetic or logical problems, or generate responses that are seemingly obvious yet erroneous. And unlike humans, the models sometimes produce outputs that are outlandish and/or realistic yet fake (e.g., properly formatted citations to plausible-sounding journal articles that do not actually exist). The current state of research does not provide a clear methodology for guiding LLMs to minimize these inappropriate, false, or misleading outputs. Further compounding this challenge is the fact that LLMs by default do not have the capability to accurately attribute the sources underlying the information that they provide, and it depends on which layer of the foundation model [62] supply chain the clinical applications are built on top of. Such lack of attribution compounds the practical challenge of double-checking the information provided by any given LLM.

Second, scientific understanding of the mechanisms by which LLMs generate seemingly intelligent responses is still in its infancy [63]. For instance, if a user shares feelings of intense despair or mentions self-harm, an LLM that generates responses based on learned patterns rather than a real understanding of context or emotion might fail to accurately identify the urgency or respond appropriately. This lack of genuine empathy and understanding may perpetuate negative user experiences or intensify feelings of distress. Thus, while LLMs can aid mental health services, they must be used with robust monitoring systems and cannot replace professional intervention. Such monitoring systems may in turn require the oversight of clinical professionals, potentially providing additional roles and responsibilities for mental health clinicians.

Third, LLMs inherently express values in their communication, and those values might not comport with those of users. As a field, mental healthcare clinicians and researchers typically follow the principle of beneficence, which aims to promote human welfare and reduce suffering through knowledge and interventions. However, whether any particular LLM exhibits behaviors consistent with such values remains an empirical question [64].

Fourth, the potential for clinicians and researchers to become dependent on such systems is worth considering [65] as LLMs become widely adopted in the workplace. Even if such dependence does not materialize, it is likely that the requisite skills and practice of clinicians will change in the future as LLMs are integrated into clinical care settings. The manner and degree to which such change occurs is ultimately a question that will best be gauged via longitudinal study. As with any new technology or intervention, clinical trials will be invaluable in understanding where and how LLMs can be best deployed. The key elements of such trials include careful consideration of comparators, potential adverse effects, and sources of bias [66].

Finally, a suite of additional challenges – e.g., medical data imbalance and data shortage, potential data leakage and privacy implications, the need for carefully designed and evaluated prompts, the insufficient availability of open models trained across a variety of languages, the details of ethical implementation, and the difficulty of obtaining large, annotated corpora in specific medical fields – will need to be addressed in the process of incorporating LLMs into psychiatric care and research. Of specific concern are two additional facts. First, many of the health data that might be used to adapt LLMs to psychiatric contexts may fall under formal privacy protections (e.g. the Health Insurance Portability and Accountability Act, HIPAA), and ensuring such data are not subsequently revealed by the production LLM – a form of data leakage [67] – is an open problem. Second, those firms who already possess large stores of medical data such as large hospital systems and data brokerage firms that buy out data may have an inherent advantage in the use of these tools. Efforts to ensure equitable access are needed. Such challenges underscore the need for all parties engaged in mental healthcare to contemplate carefully how best to deploy LLMs to augment individual well-being.

### Establishing responsible guardrails for the use LLMs in psychiatric care and research

In the context of AI deployment, the field of Responsible AI (RAI) has examined the ethical use of AI systems in various contexts, with many industry and academic researchers suggesting design principles and guidelines for others to follow. In practice, following these principles requires a significant amount of human oversight and investment to adhere to these principles. For instance, this includes impact assessments, a deep analysis of how AI affects stakeholders, and considering societal, cultural, and ethical implications, and it requires implementing human checks and controls to guide AI behavior, ensuring alignment with ethical standards and societal norms [68].

Perhaps the most critical activity of RAI human oversight is called ‘red-teaming’ [69]. The term, borrowed military strategy, describes the practice of adopting an adversarial approach to challenge and improve strategies, plans, and systems by identifying vulnerabilities and mitigating potential harms an opponent might exploit. This concept has been subsequently applied to technology, especially in cybersecurity. With the recent boom in generative AI technologies, AI red-teaming has gained considerable traction and attention as a method of ensuring AI safety, with AI companies jumping to hire professional and volunteer red-teamers (e.g., the most recent example being OpenAI’s announcement in February 2024 of Sora, a text-to-video engine that is currently available only to red-teamers).

The range of practices for AI red-teaming varies from open-ended to targeted and from manual to automated. For example, GPT-4 went through iterative rounds of red-teaming with the help of domain experts [70]. In another example, a Multi-round Automatic Red-Teaming (MART) method was used to enhance the scalability and safety of LLMs by iteratively fine-tuning a target LLM against automatically generated adversarial prompts, reducing safety violations by up to 85% without compromising performance on non-adversarial tasks [71].

Despite AI red-teaming efforts focused on computing and ethics, the omission of psychiatric-specific concerns represents a significant gap, hindering the responsible use of LLMs in psychiatric care and research. For psychiatric applications of red-teaming, key considerations include scoping intended application scenarios, selecting a diverse group for testing across various demographics and experiences, defining the technical scope of what will be tested (e.g., public vs. proprietary models, safety layers, or user interfaces), and deciding on the testing methodology, whether it be organized teams or scattered volunteers, and open or targeted tasks. In developing guardrails and testing the manners in which psychiatric applications of LLMs may present challenges, the role of red-teaming for LLM-based applications in psychiatry should not be overlooked. AI in mental health must ethically align with therapeutic goals while balancing efficiency and sensitivity, respecting patient autonomy and confidentiality, and avoiding the reinforcement of stigmas. AI testing in mental health should also evaluate its impact on psychological well-being and therapeutic outcomes, focusing on data sensitivity, patient privacy, cultural adaptability, and the potential for emotional harm. There are emerging examples of efforts to establish AI safety benchmarks [72, 73], but it is worth emphasizing that implementing AI in mental health demands precision and human accountability and requires systems that adapt to individual needs and include robust mechanisms for clinician oversight to support, not replace, professional judgment.

The challenges we outline above thus necessitate the active engagement of domain experts in psychiatry to make the red-teaming of AI systems more robust, clinically sound, and safe for mental health. Such experts should include not only clinicians, but individuals with lived experience, family members, and policymakers – all of whom will be impacted by the success or failure of these models.

## Conclusion

The use of LLMs in healthcare [19], mental health, and psychiatric research remains in its infancy. These models have shown promising potential for early detection, treatment, prediction, and scientific evaluation of mental health conditions. Increasing the reliability and predictability of LLMs can enhance clinician-researcher trust and understanding, thus facilitating the successful integration of risk models into clinical care and psychiatric research. The advent of LLMs will alter the landscape of public mental health, via direct social effects likely to stem from the technology as well as via the integration of LLMs into mental healthcare and research [60]. LLMs hold significant promise for mental healthcare and research, presenting opportunities to expedite diagnostic processes, enhance patient care, and contribute to research efforts in psychiatric science. Simultaneously, the use of LLMs for personal assistance can have far-reaching implications for population mental health, both positive and negative.

Nevertheless, the deployment of these advanced AI models will bring challenges, including ethical concerns [74]. Their limitations, such as the unpredictability of outputs in some models, lack of understanding of response generation mechanisms (i.e., lack of transparency), potential for inappropriate or false outputs, and possible mismatch of values and introduction of bias, present significant hurdles. Going forward, the next steps should involve rigorous interdisciplinary dialogue and research into understanding and overcoming these challenges via the establishment of responsible guardrails. Additionally, exploring ways to fine-tune LLMs with the guidance of clinicians and to expand their accessibility to a wider range of users is essential. Finally, the profound changes LLMs might bring justify efforts that study the system-wide effects of LLMs on population mental health, including socioeconomic consequences that might subsequently alter people’s well-being. It is through these concerted efforts that we can maximize the potential benefits of LLMs in mental healthcare and research while minimizing their associated risks.

### Citation diversity statement

The authors have attested that they made efforts to be mindful of diversity in selecting the citations used in this article.
